# Handling of targeted amplicon sequencing data focusing on index hopping and demultiplexing using a nested metabarcoding approach in ecology

**DOI:** 10.1038/s41598-021-98018-4

**Published:** 2021-09-30

**Authors:** Yasemin Guenay-Greunke, David A. Bohan, Michael Traugott, Corinna Wallinger

**Affiliations:** 1 Applied Animal Ecology, Department of Zoology, University of Innsbruck, Technikerstraße 25, 6020 Innsbruck, Austria; 2 Institute of Interdisciplinary Mountain Research, IGF, Austrian Academy of Sciences, Technikerstraße 21a, 6020 Innsbruck, Austria; 3 Agroécologie, AgroSup Dijon, INRAE, Université Bourgogne Franche-Comté, 21000 Dijon, France

**Keywords:** Biological techniques, Computational biology and bioinformatics, Ecology, Molecular biology

## Abstract

High-throughput sequencing platforms are increasingly being used for targeted amplicon sequencing because they enable cost-effective sequencing of large sample sets. For meaningful interpretation of targeted amplicon sequencing data and comparison between studies, it is critical that bioinformatic analyses do not introduce artefacts and rely on detailed protocols to ensure that all methods are properly performed and documented. The analysis of large sample sets and the use of predefined indexes create challenges, such as adjusting the sequencing depth across samples and taking sequencing errors or index hopping into account. However, the potential biases these factors introduce to high-throughput amplicon sequencing data sets and how they may be overcome have rarely been addressed. On the example of a nested metabarcoding analysis of 1920 carabid beetle regurgitates to assess plant feeding, we investigated: (i) the variation in sequencing depth of individually tagged samples and the effect of library preparation on the data output; (ii) the influence of sequencing errors within index regions and its consequences for demultiplexing; and (iii) the effect of index hopping. Our results demonstrate that despite library quantification, large variation in read counts and sequencing depth occurred among samples and that the sequencing error rate in bioinformatic software is essential for accurate adapter/primer trimming and demultiplexing. Moreover, setting an index hopping threshold to avoid incorrect assignment of samples is highly recommended.

## Introduction

Targeted amplicon sequencing (TAS) or targeted analysis sequencing is a method which addresses the sequencing of specific amplicons and genes. The approach is technologically rooted in next-generation sequencing (NGS), also called high-throughput sequencing (HTS) or massively parallel sequencing and offers the possibility to read millions of sequences in one sequencing run. The rapid evolution of NGS technology with constant increases in sample numbers, data output per sequencing run and associated decreases in costs, has led to this approach becoming widely used in various areas of research. With epigenome, genome and transcriptome sequencing, NGS extends over a wide field, regardless of the different biological disciplines (e.g., botany, ecology, evolutionary biology, genetics, medical sciences, microbiology, zoology, etc.)^1–8^. In addition to the use of NGS runs in studies to research gene regulation and expression, the characterization of mRNA during transcriptome analyses, the development of molecular markers and genome assembly, another possible application in the context of TAS is the investigation of genetic variation. There is a large range of possible TAS applications including variant detection and tumour profiling in cancer research, the detection of somatic mutations or those associated with susceptibility to disease, new findings in the field of phylogeny and taxonomy studies or the discovery of useful genes for applications in molecular breeding^2,3,9,10^. In the field of environmental sciences, TAS is becoming increasingly important, as it facilitates the assessment of the taxonomic composition of environmental samples with the help of metabarcoding approaches such as environmental DNA (eDNA) based biomonitoring or food web studies^11–13^.

Although NGS-based TAS is a powerful approach, different errors and biases can be introduced in such data sets. Sequencing errors have already been documented in medical studies, wherein factors such as sample handling, polymerase errors and PCR enrichment steps were identified as potential biases^14,15^. Similarly, other factors such as the variation in sequencing depth between individual samples, sequencing errors rates and index hopping can also play an important role within the analysis of NGS data. The difficulty is that there are currently no general standards requiring detailed reports and explanations to correct such potential errors, and very few studies have addressed this issue. Moreover, there is ever increasing access to NGS platforms, provided by sequencing companies, core facilities and research institutes^16,17^. NGS services often only provide the sequencing data while general information on the particular NGS run, demultiplexing-efficiency of individual samples and other relevant parameters are usually not passed on. The lack of such information and of a precise description of bioinformatic data processing makes it difficult to assess how the respective NGS run and the subsequent data processing went, which in turn complicates the comparison of results from different studies. Here, we show that specific aspects of library and data preparation have a critical influence on the assignment of sequencing results and how these problems can be addressed using a carabid beetle trophic data set as a case study system.

Currently, a widely used approach to study large sample numbers is the analysis of pooled samples, by combining DNA from multiple individuals into one sample of the NGS library, thereby excluding the opportunity of backtracking specific sequences to an individual sample (no individual tagging)^18–20^. In ecological studies (e.g., in biodiversity research and functional ecology), the analysis of such pooled samples may then lead to a decreased estimate of the diversity of the identified species compared to an individual-based analysis^21^. Aside from the potential loss of information, pooled samples make it impossible to assign a given sample to its specific collection site and thus, the ability to refer to habitat related differences. For individual-level analyses, the ‘nested metabarcoding approach’^22^ offers a promising solution to problems of complexity and cost. It is both a cost-efficient NGS protocol and one that is scalable to hundreds of individual samples, making it ideal for any study that relies on high sample numbers or that analyses samples which need to be tagged individually, such as in the medical field for patient samples. Using the nested metabarcoding approach, each sample is tagged with four indexes defining a sample. The presence of sequencing errors within the index region can complicate the demultiplexing process and thus the identification of the sample affiliation of individual reads. For a precise assignment of reads to each sample using the index combinations, sequencing errors must be considered in the analysis in order to be able to assign a maximum number of reads.

Besides sequencing errors within the different index regions that renders the read assignment difficult, a well-known, but at the same time often ignored problem is ‘index hopping’. This phenomenon, also called index switching/swapping, describes the index mis-assignment between multiplexed libraries and its rate rises as more free adapters or primers are present in the prepared NGS library^23,24^. Illumina therefore differentiates between combinatorial dual indexing and unique dual indexing. Special kits are offered with unique dual index sequences (set of 96 primer pairs) to counter the problem of index hopping and pitfalls of demultiplexing. This is an option for low sample numbers, as these can still be combined with unique dual indexes (UDIs). If several hundred samples are to be individually tagged in one run, it can be difficult to implement unique dual indexing due to the high number of samples and for cost reasons. Here, the nested metabarcoding approach offers a convenient solution for analysing a large number of individual samples at comparatively low costs. However, it is important to be careful regarding index hopping since more indexes are used in the nested metabarcoding approach than for pooling approaches. For instance, in silico cross-contamination between samples from different studies and altered or falsified results can occur if a flow cell lane is shared and the reads were incorrectly assigned. Even where samples are run exclusively on a single flow cell, index hopping may result in barcode switching events between samples that lead to mis-assignment of reads.

For library preparations of Illumina NGS runs, two indexes are usually used to tag the individual samples (dual indexing)^25^. Illumina offers the option to do the demultiplexing and convert the sequenced data into FASTQ file formats using the supplied 'bcl2fastq' or 'bcl2fastq2' conversion software tool^26^. This demultiplexing is a crucial step, as it is here that the generated DNA sequences are assigned to the samples. In most cases, the data is already provided demultiplexed after the NGS run by the sequencing facility, especially if runs were shared between different studies/sample sets. Researchers starting the bioinformatic analysis with demultiplexed data assume that the assignment of the sequences to samples was correct. Verifying this is extremely difficult because the provided data sets lack all the information on the demultiplexing settings and, above all, on the extent of sequencing errors within indexes and index hopping. As a consequence, sequences can be incorrectly assigned to samples and, in case of a shared flow cell, even across sample sets. These steps of bioinformatic analysis are very often outsourced to companies and details on demultiplexing are seldom reported, showing that the problem of read mis-assignment has received little attention so far. However, it is known that demultiplexing errors occur and depend on various factors such as the Illumina sequencing platform, the library type used and index combinations^23–25,27–30^. The few existing studies investigating index hopping in more detail give rates of 0.2–10%^24,31–34^. This indicates the importance of being able to estimate the extent of index hopping for a specific library. The problem of sequencing errors within indexes and index hopping can become particularly significant if, due to the large number of individual samples, libraries were constructed with two instead of one index pair, such as it is the case in the nested metabarcoding approach^35^. Then, one is inevitably confronted with the effect of sequencing errors and index hopping on demultiplexing and subsequently on the data output.

After each NGS run, the combination of computational power and background knowledge in bioinformatics are needed to ensure time-efficient and successful data analysis^36^. But even for natural scientists with considerable bioinformatic experience, there is a lack of know-how or even rules-of-thumb in this still nascent field. It is well known that specific decisions have a marked impact on the outcome of a study, with both the sequencing platform and software tools significantly affecting the results and thereby the interpretation of the sequencing information^37^. Knowledge of the individual data processing steps, such as for the demultiplexing, is also often missing or poorly described. Information on how to minimize data loss within the individual steps for data preparation of the NGS data is also mostly not explained. Given this lack of detail, it is a challenge to understand what was done during sample processing and data analysis, and impossible to compare the outcomes of different studies. To date, published NGS studies, such as TAS or DNA metabarcoding studies, are difficult to compare or evaluate because of the lack of this essential information on data processing. This is particularly important as NGS is increasingly being done by external service providers. As a consequence, there is a pressing need for comprehensive protocols that detail the aspects that need to be considered during analysis.

Using a case study on the dietary choice of carabid beetles (Coleoptera: Carabidae) in arable land, we detail a comprehensive protocol that describes an entire workflow targeting *ITS2* fragments, using an Illumina HiSeq 2500 system and applying the nested metabarcoding approach^22^ to identify those species of weed seeds consumed by carabid individuals. We demonstrate a concept that employs bioinformatic tools for targeted amplicon sequencing in a defined order. By analysing the effects of sequencing errors and index hopping on demultiplexing and data trimming, we show the importance of describing the software and pipeline used and its version, as well as specifying software configurations and thresholds settings for each TAS data set to receive a realistic data output per sample. Without this information, there is the possibility of incorrectly assigning samples or not receiving the maximum or at least a sufficient number of sequences which in turn would hamper the results.

The concept described below can be used to analyse a large number of samples, here to identify food items on species-specific level, and to address the possible problems that may arise in NGS data processing. We identify problems to overcome and potential solutions by examining: (i) the variation in sequencing depth of individually tagged samples and the effect of library preparation on the data output; (ii) the influence of sequencing errors within index regions and its consequences for demultiplexing; and, (iii) the effect of index hopping. By doing this, we highlight the benefits of a detailed protocol for bioinformatic analysis of a given data set, and the importance of the reporting of bioinformatic parameters, especially for the demultiplexing, and thresholds to be used for meaningful data interpretation.

## Materials and methods

### Sampling and DNA extraction of carabid beetles

Carabid beetles were collected for molecular gut content analyses in organic cereal fields in three Central European regions along an east–west transect, comprising the area of central Burgundy (France), Tyrol (Western Austria) and the Vienna Basin (Eastern Austria) in six fields per region at two sampling sessions, in May/June and July/August 2016. The beetles were individually forced to regurgitate and the regurgitates used for molecular diet analysis. Additional regurgitates and whole beetles that were collected in an organically managed winter wheat field in Rotholz (Tyrol, Austria) on four sampling dates from May to July in 2017 were included for analysis. The sampling of adult carabids in dry pitfall traps and their regurgitation, the storage of samples as well as DNA extraction from regurgitates and whole carabid beetles were described more in detail in the associated transect field survey from 2016^38^. Following successful regurgitation^39^, the live carabids were released back into agricultural fields.

### Sample information and preparation for NGS

Many of the collected carabid species are classified as omnivores, feeding on both animal and plant/seed prey. In an initial screening, DNA extracts of dietary samples were tested for the presence of DNA of different animal prey types and plant DNA via diagnostic multiplex PCR^38^. Only samples that tested positive for plant DNA (with primers targeting the *trn*L intron: c-A49325 and h-B49466^40^) were chosen for NGS library preparation. Since the *trn*L intron does not allow for a species-specific identification of the consumed plant food, the *ITS2* region was selected, which has shown to provide good species resolution in plants^41^. For the present study, the nested metabarcoding approach^22,35^ was used to identify the seed food of carabids. Library preparation was conducted in the 96-well format, in this way covering 1920 dietary samples as well as positive and negative controls (n = 370). For each plate, a DNA extract from the common dandelion (*Taraxacum officinale*) was used as positive control and a molecular grade water control to test for contamination during PCR. The dietary samples not amplifying *ITS2* fragments in the two consecutive PCRs were randomly distributed among the plates. All control samples, common dandelion, molecular grade water and dietary samples without any *ITS2* amplicon, were not sent for sequencing (Fig. S2). Accordingly, the 370 index combinations of these control samples served as quality controls for index hopping during the bioinformatic analysis.

**Figure 1:**
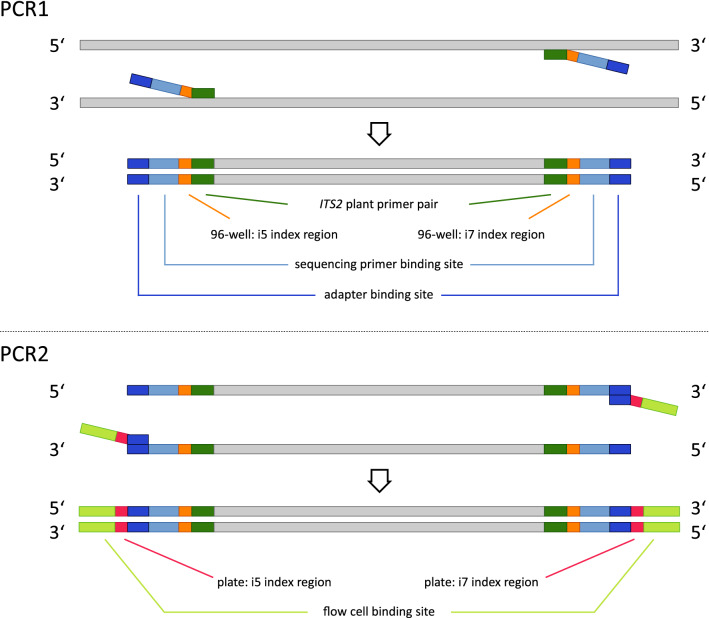
NGS library preparation for the applied targeted amplicon NGS and nested metabarcoding approach comprising two consecutive PCRs. In PCR1, DNA fragments are amplified to which the following is attached: primers targeting the genomic region *ITS2*, indexes to identify the 96 samples per plate (sample specific combination of i5 and i7 indexes), sequencing primer binding site, and the adapter binding site. In PCR2, additionally, specific combinations of indexes of i5 and i7 indexes for each plate are added as well as the binding site for the flow cell of the Illumina platform.

In a first step, a general plant primer pair was designed on the basis of previous plant primers targeting the *ITS2* gene locus^41^. Additionally, the primers included overhangs for different indexes and nucleotides of the binding region for Illumina Nextera adaptors (Table S1). To generate pre-libraries that obey the Illumina sequencing workflow, PCRs were performed (PCR1, Fig. 1), amplifying fragments of *ITS2* by combining the UniplantR (5′-CCCGHYTGAYYTGRGGTCDC-3′)^41^ reverse primer with the newly designed forward primer UniPlantF2 (5′-GGCACGYCTGYBTGG-3′). To tag each sample individually during this first PCR, Illumina Nextera DNA indexes were combined for the plates by taking the extended forward primer with different i5 indexes per lane (A-H: [E/H/N/S] 513, 515–518, and 520–522) and reverse one with i7 indexes per column (1–12: [H/N] 716, 718–724, and 726–729)^42^ (Table S1). All HPLC-purified oligonucleotides used for library preparation were purchased in individual tubes from Eurofins Genomics Germany GmbH (Ebersberg, Germany). Each 20 μl volume for the PCR included 6 μl DNA extract, 1 μl bovine serum albumin (BSA) (10 mg/ml), 10 μl 2 × Type-it Mutation Detect PCR Kit (Qiagen, Hilden, Germany), 2 μl molecular grade water, and 0.5 μl for each primer (10 μM). The thermal cycling scheme was as follows: 5 min at 95 °C followed by 40 cycles of 30 s at 95 °C, 90 s at 55 °C, 60 s at 72 °C and a final extension for 5 min at 72 °C. All PCR products were visualized using the automated capillary electrophoresis system QIAxcel with the QIAxcel DNA Screening Kit (both: Qiagen, Hilden, Germany), the method AM320 and an injection time of 30 s. Results were scored with the software QIAxcel ScreenGel v1.6.0.10 (Qiagen, Hilden, Germany). Samples showing the expected fragment length with a signal above 0.1 relative fluorescent units (RFU) were deemed to be positive.

In a second PCR, all samples within a 96-well plate were additionally indexed by adding the Nextera adaptors (PCR2). Each of the 24 plates received its individual index combination comprising the i5 indexes [E/H/N/S] 502, 503, 505–508 and i7 indexes [H/N] 702–705^42^, finally resulting in 2,290 individual index combinations (Fig. S1). The 25 μl reaction volume of each PCR2 contained 5 μl template PCR product from PCR1, 1 μl bovine serum albumin (BSA) (10 mg/ml), 12.5 μl 2 × Type-it Mutation Detect PCR Kit (Qiagen, Hilden, Germany), 1.5 μl molecular grade water, and 2.5 μl of adapter 1 and 2 each (10 μM) (see Table S2 for adapter sequences). The thermal cycling scheme was as follows: 5 min at 95 °C followed by 8 cycles of 30 s at 95 °C, 30 s at 55 °C, 60 s at 72 °C and a final extension for 5 min at 72 °C. The separation and visualization of the PCR products on a QIAxcel system was done as described above. All individual dietary samples were then pooled into one sample per plate (‘plate sample’ Fig. S2), with the control samples undergoing no further analysis. The specific volume of each dietary sample in this pool depended on its RFU value: 25 μl (whole) reaction volumes were taken from samples with an RFU between 0.1 to 4.0, 10 μl from those with an RFU larger than 4.0 up to 6.0, and 5 μl from those ones with an RFU larger than 6.0. This resulted in 24 plate samples that were purified with the magnetic bead capture kit SPRIselect (method: left side selection) (Beckman Coulter, Inc., Brea, U.S.A.) following the manufacturer’s instructions, before sending them for sequencing.

In the nested metabarcoding approach, the library consists of an inner (ID for one of the positions in the 96-wells) and an outer (plate ID) pair of index combinations (see Fig. 1). The outer pair of indexes corresponds to the standard unique dual index (UDI) combination, which is recognized by the sequencer as an index, while the inner index combination is also sequenced as part of the read. Thus, the 8 nucleotides of the inner index region are sequenced before the *ITS2* plant primer and are at the beginning of each read of the data output, since they are not recognized as index by the software of the sequencer and are therefore not demultiplexed.

### Next-generation sequencing run

Given the fragment size of the *ITS2* amplicon for plants (~ 230 bp without primer sequences) and the need to guarantee a broad coverage of amplicons per sample, the Illumina HiSeq 2500 system (Illumina, San Diego, USA) was chosen as the sequencing platform. The sequencing run was performed as follows: paired-end run (2 × 250 bp) under rapid run mode using a two-lane rapid flow cell (HiSeq Rapid SBS Kit v2 (500 cycles): Illumina, San Diego, USA), exclusively filled with the described plant amplicons of the carabid diet, to ensure that no cross-contamination from samples of other studies do occur. Plate samples 1 to 12 were loaded onto lane 1, and samples 13 to 24 onto lane 2. Each lane contained samples of different carabid species and sampling sites to avoid any selection bias. Sequencing was conducted at the Vienna BioCenter Core Facilities (VBCF) (Vienna, Austria).

### Bioinformatic analysis of NGS data and its visualisation

**Figure 2:**
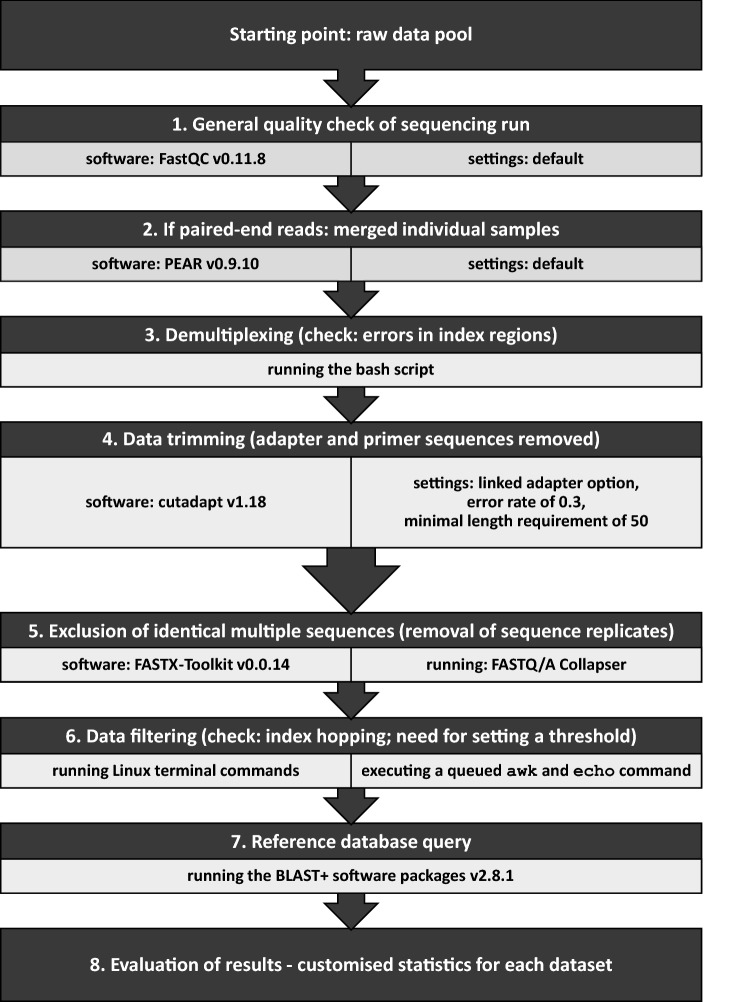
Diagram of the eight data processing steps for the evaluation of the applied nested metabarcoding approach.

The bioinformatic workflow for the assessment of the carabids’ diet comprised eight data processing steps (Fig. 2). As a first step, the paired-end sequencing raw data files obtained from the 24 plate samples were checked by the software FastQC v0.11.8^43^ to get an overview of the data quality (step 1). The quality control checks of plate samples allowed the paired-end reads to be directly merged using the software PEAR v0.9.10^44^, applying default settings (step 2). Then, demultiplexing of the plate samples to the 2,290 individual samples (370 control and 1,920 dietary samples) was done. The outer indexes, which were the plate IDs in the present case, were processed with the Illumina software ‘Illumina Experiment Manager v1.16.1’ (IEM) and assigned by Illumina’s ‘bcl2fastq2 Conversion Software v2.20.0’, accepting single-nucleotide mismatches as standard. In the nested metabarcoding approach, a second pair of indexes is inserted. The eight nucleotides of this inner index pair, representing the sample ID and that were added to the *ITS2* primers in PCR1, were examined more closely in order to optimize the demultiplexing of the individual samples. The goal here was to obtain as many sequences per sample as possible and at the same time not to introduce any errors. Instead, a detailed analysis of the positions of potential sequencing errors was done that allows reliable error correction, by comparing all pairs of used i5 and, separately, i7 indexes from the Illumina set. For the index sequences used here, this analysis showed that any single mismatch between read and inferred index (in any of the eight positions of the index) could still be tolerated to be able to correctly assign the read to the respective sample. Likewise, multiple errors in positions three to six of the forward (i5-based) indexes or in positions three to five of the reverse (i7-based) indexes could be assigned to the closest matching index, provided that the flanking positions matched. This analysis step was done with the help of a specially written bash script (step 3, see supplements for more details). Applying this script, only one dietary sample had to be excluded for further analysis, as no sequences could be assigned to it. Afterwards, the merged reads were trimmed by removing the primer sequences using cutadapt v1.18^45^, with the linked adapter option, an error rate of 0.3 (see “Analysis of sequencing errors for demultiplexing and data trimming” section results part) and a minimal length requirement of 50 (step 4). Prior to a local BLAST search, replicates of identical sequences were removed to obtain a single unique sequence per replicate. In this step, the number of copies per specific sequence within a specific dietary sample was counted by running the application FASTQ/A Collapser of the FASTX-Toolkit v0.0.14^46^ (step 5). The number of reads per control sample served as the basis for setting a threshold for index hopping. Samples with more than 280 copies per unique sequence, the defined index hopping threshold, were removed by executing a queued awk and echo command at the Linux terminal (see “Handling of index hopping: Is there a need to set a threshold?” section results part) (step 6). To run BLAST+ software packages v2.8.1 locally in the most efficient way, the nt database (ftp://ftp.ncbi.nlm.nih.gov/blast/db/, status: 25.7.2017) was downloaded. All BLAST+^47^ searches were performed on the high performance compute cluster LEO4 of the University of Innsbruck (step 7) and the plant species in the diet of each carabid were identified (step 8, not presented here). Statistical analysis using Pearson correlation and data visualisation was conducted in R version 3.6.3^48^ using the packages “ggplot2”^49^ and “cowplot”^50^.

## Results

### General data output

In the present study, 24 96-well plates with a total of 2,290 samples (dietary and control samples) were analysed in two consecutive PCRs during library preparation, of which 1,920 samples were pooled and sent for NGS. The dandelion *ITS2* amplicons were detected in all positive controls and all water controls were clean, which indicates successful amplification but also makes DNA cross-contamination extremely unlikely. Paired-end data were generated for each plate sample, on both lanes of the NGS run. About 2 × 78 million reads were amplified for lane 1 and 2 × 82 million reads for lane 2. Before merging (step 2) and demultiplexing (step 3) the paired-end data, sequence read counts ranged from 5,872,476 to 7,855,719 reads (unmerged, R1 files: forward reads) for plate 1 to 12 (lane 1) and from 5,505,974 to 9,187,035 reads for plate 13 to 24 (lane 2) (step 1). For the respective steps 2 and 3, the data loss was different for each lane as well as for the plate samples within the lanes. After the merging step, the range of the plate samples went down to values between 5,801,292 and 7,775,498 reads in lane 1 (0.7–1.3% data loss) and between 5,415,150 and 9,052,475 in lane 2 (1.0–1.8% data loss). The data loss during the merging step was mainly based on a cleaning process. The *ITS2* primers used do not only bind to plant DNA but also to non-target organisms like fungi and bacteria^41^. The resulting fragments, however, could be differentiated by amplicon size and were therefore removed in this step. Because of this, the data loss during demultiplexing was higher than the one in step 2, even though we used the adapted bash script in order to keep the loss as small as possible. This resulted in read counts of 5,801,292 to 7,775,498 for lane 1 (4.0–6.2% data loss) and 5,415,150 to 9,052,475 for lane 2 (13–23.2% data loss) (see Table S3 for more details about read counts per plate sample). After merging and demultiplexing (steps 2 + 3), about 63 million quality-filtered sequence counts of paired reads were obtained for lane 1 and 59 million for lane 2, whereby a data loss of 19.2% for lane 1 and 28.0% for lane 2 could be calculated.

### Sequencing depth for each individual sample and its variability

**Figure 3:**
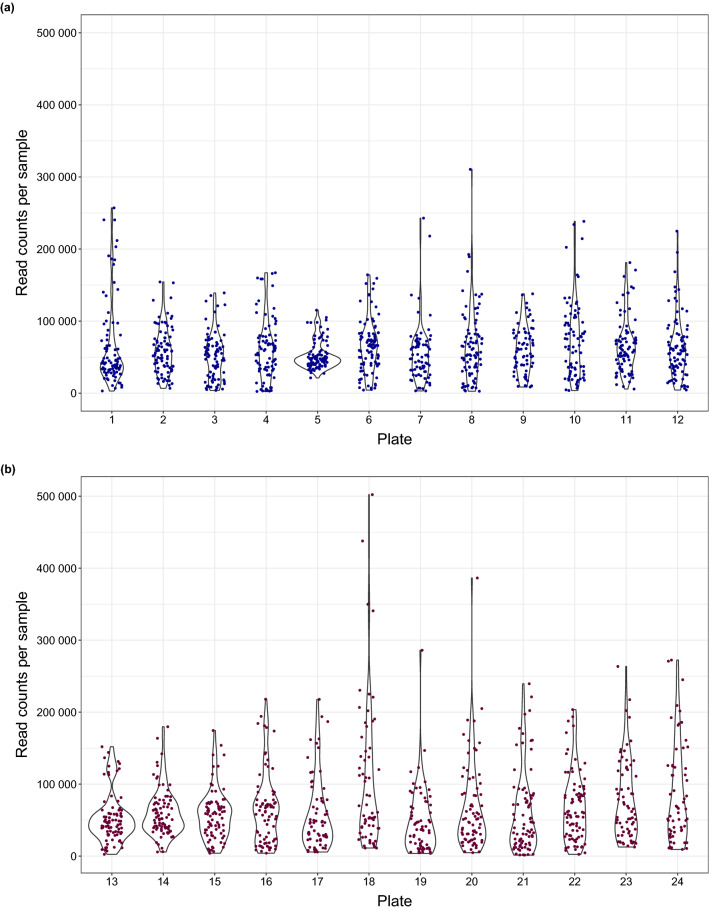
Read counts per sample after adapter trimming, shown for the plates (**a**) 1 to 12 of lane 1 and (**b**) 13 to 24 of lane 2, dots represent the individual dietary samples in lane 1 (n = 1028) and lane 2 (n = 892).

**Figure 4:**
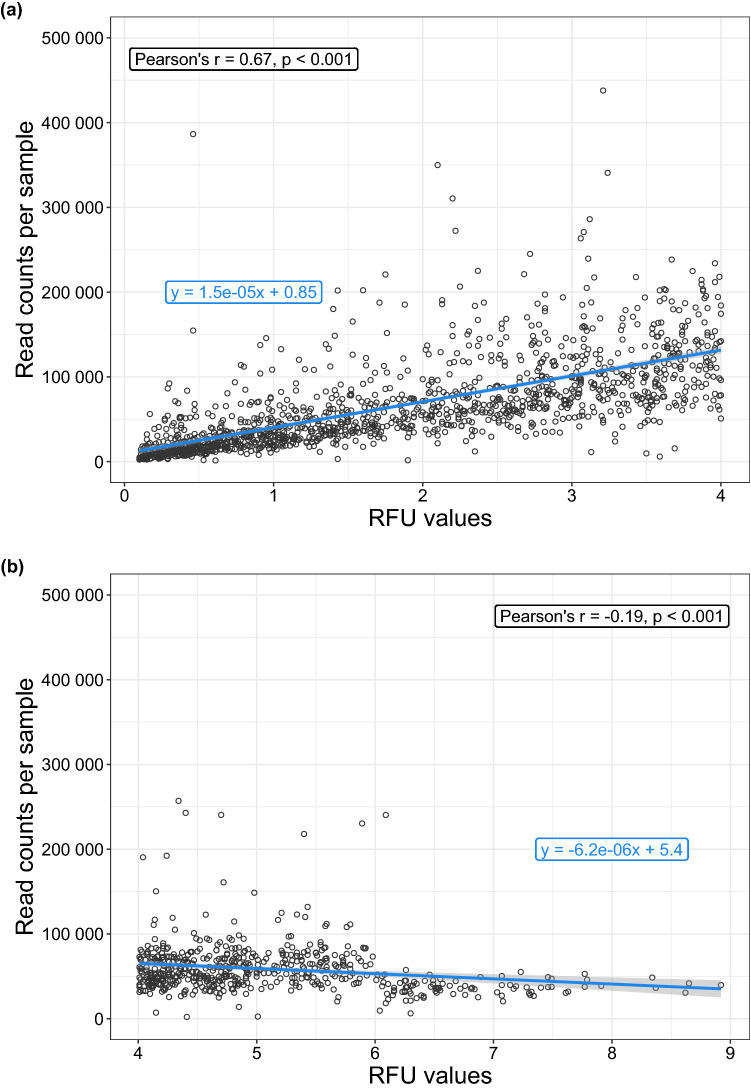
Influence of library preparation on sequencing depth visualized by plotting the outcomes of the NGS analysis against the signal strength of the products after library preparation that result from the two consecutive PCRs: that is the read counts per dietary sample (NGS) against the relative fluorescent units (RFU) of the automated capillary electrophoresis system QIAxcel for (**a**) small amounts of amplicons (≤ 4.0 RFU) and (**b**) larger ones (> 4.0 RFU). The individual dietary samples are represented by the circles.

Approximately equally distributed read counts between samples were expected from the pooling of individually labelled dietary samples for the NGS run, depending on the signal strength of PCR2. However, there was huge variation in the read counts when comparing all dietary samples with each other (Fig. 3a,b). Likewise, the variability of sample read counts between the different plates was high. Plate 5, as an example, had only a few samples with higher or lower read counts than the average, and a low variation in read count data, leading to maximum differences in read counts of 5.4 times between samples. By contrast, when comparing dietary samples of plate 18, the lowest to highest read counts differed by a factor of 45 times. Across all 1920 dietary samples, there was a maximum difference of approximately 360 times as many sequences between the samples with the highest and lowest sequence number. The potential effect of the ‘equimolar’ pooling during library preparation on the observed variability in read counts was tested by calculating Pearson's correlation coefficient (Fig. 4) for the relationship between read counts and the RFU value of each sample. This correlation was negligible over the entire data set (*r* = 0.28, *p* < 2.2 × 10^–16^).

### Analysis of sequencing errors for demultiplexing and data trimming

**Figure 5:**
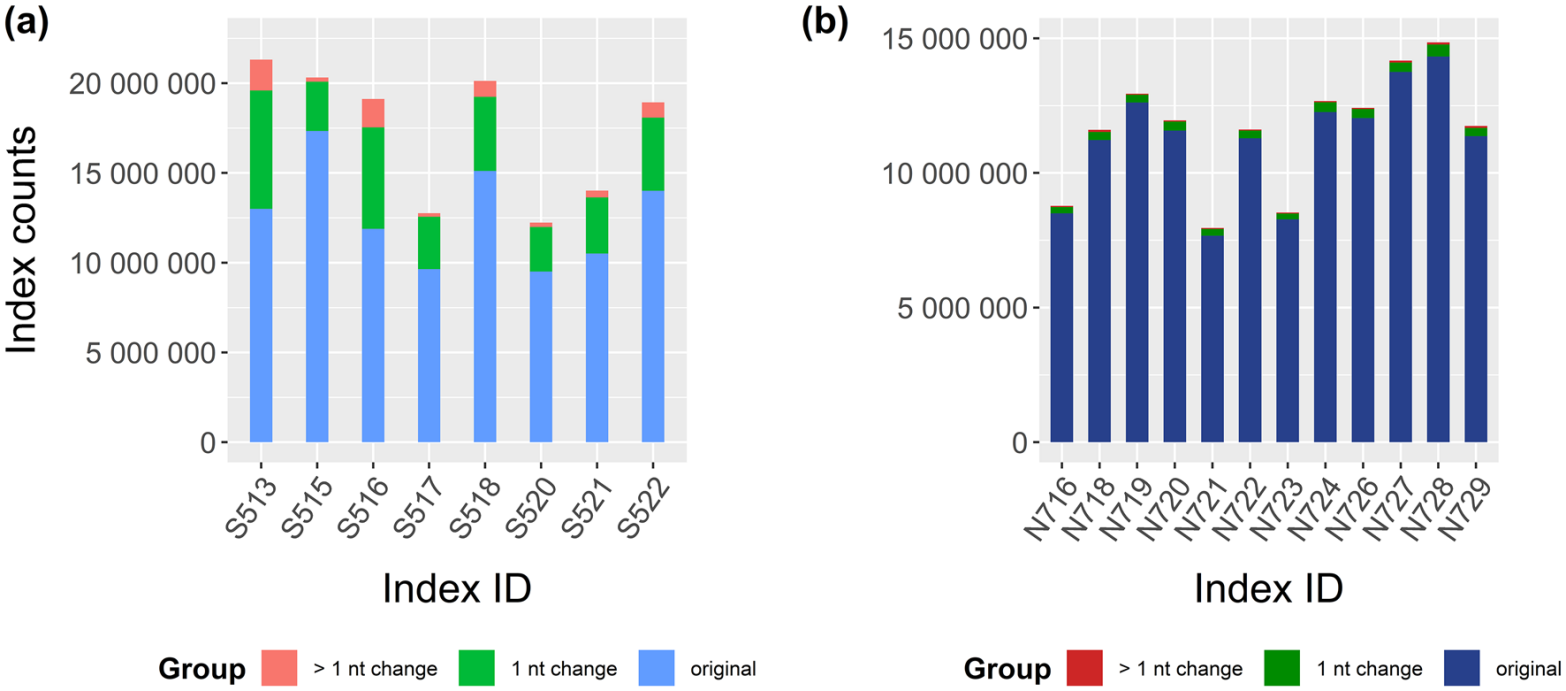
Sequencing error proportions for Nextera index regions (**a**) (S5xx) and (**b**) (N7xx) indexes; counting reads either containing more than one nucleotide differences (> 1 nt change), exactly one nucleotide difference from the original eight nucleotide index region (1 nt change) or no sequencing error within index region (original). Analysed reads were obtained from merged plate data.

**Figure 6:**
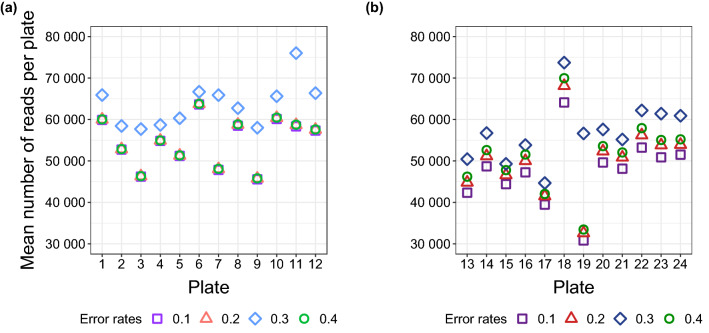
Comparison of changed error rates on the number of trimmed reads for plate samples of (**a**) Lane 1 and (**b**) Lane 2 by using the software cutadapt.

Sequencing errors can affect data outcomes during demultiplexing when index regions are affected and during PCR-based library preparation and cluster generation on the flow cell for Illumina’s sequencing by synthesis (SBS) technology. In the nested metabarcoding approach^35^, it is necessary to demultiplex plate samples to individual ones. We first checked the merged reads for sequencing errors in the index region. The proportion of reads with zero index mismatches in read 1 (i7 sequences) was 95% for lane 1 and 94% for lane 2, and in read 2 (i5 sequences) it was 68% and 48%, respectively (Table 1). It is standard practice to correct indexes with just one error and to assign them reliably to the samples. The proportion of reads with a single mismatch, which could be safely corrected, was 2.5% and 2.7% for lanes 1 and 2 in read 1 and was 19.6% and 26.5% in read 2 (Fig. 5). The largest proportion of errors in merged reads occurred in the i5 index regions with a single misread nucleotide in 23% of sequences (Fig. 5a), while it was only 3% for i7 indexes (Fig. 5b). Four percent of the i5 indexes had more than one misread nucleotide and 0.06% of i7 indexes (Fig. 5a,b). Using the new bash script, additional reads were correctly assigned to the samples, i.e., 0.1% reads per lane for read 1 and 3.6% (lane 1) and 5.3% (lane 2) for read 2. The proportion of mismatches and thus the sequencing quality varied significantly among index sequences (*p* < 0.001). Our specifically adapted demultiplexing setup allowed, on the one hand, a good assignment of reads to individual samples and, on the other, the achievement of an average of 99.94% and 99.79% of reads per dietary sample for lane 1 and 2 (Table 1), compared to 96.3% and 94.5% using the standard 1-mismatch-maximum approach. In addition to the check of the index regions, we tested the effect of the ‘maximum error rate’ parameter on data trimming (i.e., removing primer dimers and cutting off adapter, index, and primer sequences) with cutadapt. The software was run with the maximum error rates of 0.1, 0.2, 0.3 and 0.4 for each sample individually. Results were evaluated by comparing the read numbers in the output files of trimmed and non-trimmed reads for each of the four tested error rates. We found that the trimming of every sample was best with a maximum error rate of 0.3, because a maximum of cleaned sequences was achieved. This was also demonstrated by calculating the number of trimmed reads per plate (Fig. 6a,b), whereby differences between the maximum error rates of 0.1, 0.2 and 0.4 were more obvious for the plates of lane 2.

**Table 1:** Comparison between indexes of raw data sequences and merged, demultiplexed sequences for each plate.

Sequencing lane and plate	% of forward (S5xx) indexes without sequencing errors	% of reverse (N7xx) indexes without sequencing errors	% of kept sequences per plate after demultiplexing	Average % of data loss per sample after demultiplexing
**Lane 1**				
Plate 1	64.8	95.7	94.2	0.06
Plate 2	68.4	95.8	94.6	0.06
Plate 3	70.9	95.7	94.3	0.06
Plate 4	70.6	95.7	94.9	0.05
Plate 5	69.1	96.2	95.1	0.05
Plate 6	71.0	95.6	94.7	0.05
Plate 7	64.7	94.7	93.5	0.07
Plate 8	67.4	93.9	94.4	0.06
Plate 9	68.2	94.0	93.6	0.07
Plate 10	68.8	94.1	93.7	0.07
Plate 11	65.8	94.4	92.9	0.07
Plate 12	68.1	94.2	94.7	0.06
**Lane 2**				
Plate 13	49.5	93.9	79.0	0.22
Plate 14	45.3	94.0	75.9	0.25
Plate 15	46.4	93.3	79.1	0.22
Plate 16	52.8	93.8	80.5	0.20
Plate 17	45.0	92.0	77.9	0.23
Plate 18	47.3	93.7	77.0	0.24
Plate 19	48.0	95.1	85.8	0.15
Plate 20	47.9	94.8	79.3	0.22
Plate 21	52.0	94.8	81.9	0.19
Plate 22	49.8	94.9	80.0	0.21
Plate 23	46.0	95.3	78.4	0.23
Plate 24	46.4	94.9	80.8	0.20

The number of reads is given in percent (%), with a specification of: (i) how many reads are present without any sequencing errors in the indexes (column 1 + 2), (ii) how many reads can still be assigned to samples when running the demultiplexing (bash) script (column 3) and (iii) how many reads per sample were lost on average due to the demultiplexing script used (column 4).

### Handling of index hopping: Is there a need to set a threshold?

**Figure 7:**
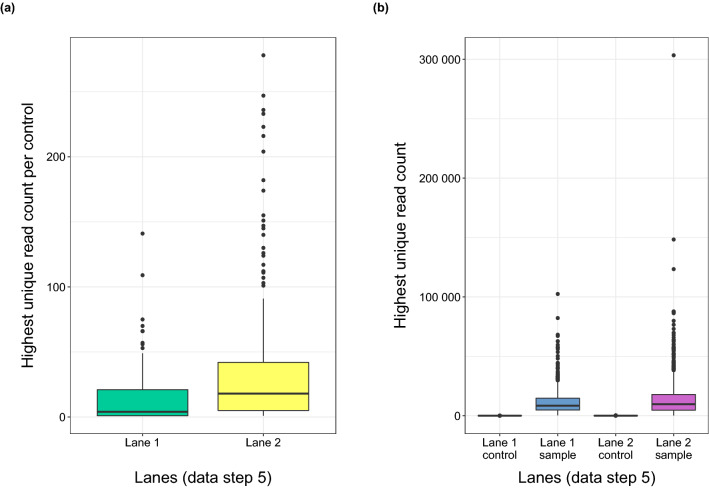
(**a**) Boxplots showing the highest unique read count (read number of highest abundant sequence within one control after reduction filtering step (5)) for each of the control samples (n = 370) of lane 1 and 2. According to the largest data point of 280 read counts for lane 2, this value was used as index hopping threshold. (**b**) Comparison of the highest unique read count between control samples (n = 370) and dietary samples (n = 1920) for both lanes; boxplots of controls are displayed larger in (**a**).

Given that the control samples were not sent for NGS, their index combinations should have been absent in the data outcomes after the demultiplexing step of the plate samples. Read counts for all 370 control samples were detected, demonstrating that index hopping had occurred. We therefore defined an index hopping threshold. The number of reads for each control sample was checked and compared to sample read counts after demultiplexing (step 3), adapter trimming (step 4) and removal of identical multiple sequences (step 5). The read counts of control samples varied between 14 and 1648 reads after demultiplexing and between 14 and 1592 reads after adapter trimming (Fig. S3a). The comparison between read counts of dietary samples, ranging from 1455 to 504,616 reads after demultiplexing (step 3) and 1401 to 502,232 reads after adapter trimming (step 4) (Fig. S3b). The read counts of control samples indicated that setting a threshold for index hopping would not lead to any essential data loss—that there is no drop of entire samples. After replicates of identical sequences were removed and the number of copies per sequence recorded (step 5), the read number of the most abundant sequence type within one control sample was used as a threshold for index hopping. Taking the highest unique read count of control samples, this gave a data value of 280 reads (lane 2) per unique sequence as the index hopping threshold (Fig. 7a). Sequences with less than or equal to 280 reads were deleted from each individual sample. As a result, only two samples no longer showed any sequences (one sample after step 3 and another one after step 6) and 1918 samples (99.90%) had further sequences with higher read counts than 280, which remained for data analysis. This is also reflected by comparing highest unique read counts of control and dietary samples (Fig. 7b). This comparison showed that the highest read counts of most dietary samples were not affected by the index hopping threshold of 280 reads, resulting in no loss of entire dietary samples except the mentioned two.

## Discussion

This work builds a workflow concept for targeted amplicon sequencing, using the trophic interactions between carabids and seed species as a case study. In this context, we show the possibility of using index combinations of control samples, which are not sent to the NGS and therefore can only be present in the data with the occurrence of index hopping, as a useful approach for the assessment of the extent of index hopping. The present study also demonstrates that developing and presenting a detailed description of all relevant steps of the bioinformatic analysis is essential, highlighting the effect of the setting of specific parameters, sequencing errors and index hopping which is obligatory for appropriate demultiplexing and subsequently also for data interpretation.

In the process of bioinformatic analysis, there is the possibility that individual steps in data preparation will repeatedly lead to data loss. The demultiplexing (step 3) was examined more closely, since a newly developed bash script has been run here. The aim was to be able to assign reads that showed sequencing errors within the inner index areas to the respective samples in order to keep data loss as low as possible. While the extent of data loss in this step was found to be tolerable, it may appear that it was comparably high during the merging step. This can be ascribed to the character of dietary samples such as gut content, faeces, or regurgitates where DNA is present in a semi-digested form and fragments at deviating sizes are amplified. Here, additional DNA fragments of bacteria/fungi were amplified with the use of the *ITS2* primers, albeit they were specifically tailored to target plant DNA (step 2). Using default settings, the software PEAR separated unassembled sequences below 50 nucleotides and those sequences without a minimum overlap size of 10 nucleotides, which non-target fragments did not have here (no overlap as too long), from the other sequences and saved them in a separate document. The settings and adaptation of PEAR for the merging step should be specifically adjusted to the needs for each study.

NGS platforms have the potential to detect even low-frequency DNA fragments. The prerequisite is that the DNA quantifications for the NGS libraries are precise^51^. For this it is necessary that an adequate quantity of PCR product is loaded onto a flow cell. Each lane should not be under- or over-loaded, so that the ideal cluster density on the flow cell allows accurate imaging of the individual bases per read. By pooling individual dietary samples in plate samples according to their signal strength (RFU values) an overrepresentation of individual samples was avoided. This became particularly clear in the case of samples with > 4.0 RFU (Fig. 4b). For samples with values ≤ 4.0 RFU, the entire reaction volume was mixed in in the pooling process, which usually resulted in an increase in read counts per sample (Fig. 4a). With these low starting amounts of amplicons, the amount of DNA was not reduced as a precaution. Nevertheless, the variability of read counts of dietary samples was still high (Fig. 4), both within and between the two lanes, indicating that also the flow cell loading and cluster generation can have an important influence on the results obtained.

The sequencing run with a HiSeq 2500 enabled the bases to be determined using a four-colour detection method whereas newer Illumina sequencing platforms (e.g., NextSeq 500, NovaSeq 6000) are based on a two-channel imaging approach. Illumina describes that their two-channel sequencing systems have problems when calling G bases after synthesis has terminated so that poly-G artefacts appear, which need to be removed from the reads. Otherwise, these could be mapped to reference regions with high G content. This problem, that does not occur with the 4-color channel chemistry used here, should also be relatively easy to assess with a TAS or metabarcoding approach, since the sequences of certain gene regions are already saved in the databases, and it is therefore possible to estimate whether they contain poly-G segments before the run. In general, the imaging process could have a decisive influence on de novo sequencing approaches (for both DNA and RNA sequencing) which should be considered.

General sequencing error rates are known to depend on the template (e.g., target region etc.), chemistry of library preparation and sequencing platforms, leading to a range of average error rates per base from 0.01 to 16%^52^. With the background knowledge of this large range within the average error rate per base, a very careful sequencing approach with 2 × 250 bp was chosen in this study. In doing so, the amplicon with a size of ~ 230 bp was almost sequenced twice (i.e., in both directions), guaranteeing a maximum overlap. This has the advantage that bases with a poorer quality can be easily replaced by and sequencing errors can be eliminated. The maximum read length of some Illumina sequencing platforms is limited to 2 × 150 bp. It has to be decided individually whether this is sufficient for a specific study. In the case of genome sequencing, for example, the maximum read length may be less relevant, since the reads overlap at different points due to the different amplicons distributed over the genome and sequencing errors can thereby be easily corrected. Contrastingly, in studies that deal with mutations and/or variant detections in a certain gene region, individual nucleotides can make a fundamental difference and sequencing errors should therefore be excluded as far as possible. The same holds true for metabarcoding and other approaches employing TAS. Here, it is particularly advisable to keep the overlap as large as possible so that the effect of sequencing errors can be minimized. Close to the adapter region, the chance of low-quality bases and sequencing errors rises^14,53^. Consequently, in the outer parts of the amplicon reads and also in the index regions, there is a higher probability of sequencing errors^15^ which can result in problems regarding a correct read assignment. The present data showed that errors mainly occurred in the i5 indexes compared to the i7. Following examination of the errors within index regions, an accurate sample assignment was achieved by executing an adjusted demultiplexing script, which made it possible that only two out of 1,920 dietary samples (0.1%) had to be excluded from the analysis. The setting of error rates in cutadapt (0.1 (default), 0.2, 0.3, 0.4) showed a strong impact on the data output. A value of 0.3 was found to be optimal for the current study, irrespective of plate and lane. This shows that, although recommendations are given by the default settings, it is crucial to adjust parameters during bioinformatic analysis accordingly as using the software's default settings does not necessarily result in optimal data processing.

We also investigated the possibility of in silico cross-contamination between samples using NGS technologies, the so-called index hopping. The current market leader for sequencing platforms, Illumina^27^, specifies the impact of index hopping on the data set with 0.1–2% of all reads on patterned flow cell systems using ExAmp chemistry, depending on aspects such as library type (chemistry and setup of unique dual indexing pooling combinations), its quality (clean-up for removal of free adapters) and handling (individual library storage). It has also been reported that up to 5–10% of sequencing reads were incorrectly assigned from one sample to other samples in a multiplexed pool under a HiSeq 4000 run^34^. In the data set analysed here, we lost an average of between 0.05% and 0.25% of the reads per sample per plate (see Table 1) using a HiSeq 2500 system. This indicates that the newer Illumina sequencers with ExAmp chemistry and patterned flow cells have no advantage over the older models (see HiSeq 2500 system) regarding index hopping. Barcode mis-assignment becomes especially problematic when the sequencing run is shared between different libraries (studies). It makes no difference if it is a dual indexing approach or a nested metabarcoding approach with two pairs of index combinations. In the best-case scenario of a shared flow cell, studies would include distantly related samples (e.g., fish versus insect) so that they can easily be distinguished from one another. In the worst-case scenario, though, it is possible that in silico cross-contamination between reads of different studies would occur. This risk is independent of the type of flow cell as for both random flow cells and patterned ones with nanowells the potential for index hopping may be present^27^. In order to avoid errors that can be ascribed to index hopping, we used two flow cell lanes exclusively for the dietary samples of this study and included several control samples in library preparation. To obtain the maximum yield in one sequencing run, all index combinations within a 96-well plate (12 × 8 combinations) are usually used. As the control samples (PCR positive and negative controls and samples not amplifying *ITS2* amplicons—see Fig. S2: green, blue and purple) were not sent to the NGS, the 370 index combinations of these control samples should be absent in the data set. If sequences with index combinations of these control samples occur, their proportion can nevertheless be used to determine the extent of index hopping and a threshold can be set. We can be sure that the detection of these indexes in the NGS data set cannot be attributed to a cross contamination during library preparation because all PCR negative controls were clean. Likewise, the highest unique read counts of the controls (Fig. 7) indicated that no contamination had occurred. In case of a contamination during PCR1 and PCR2, (see Fig. 1), outliers would also have to show up in the area of the dietary samples (Fig. 7b), which was not the case. We detected DNA sequences with indexes of all 370 control samples in the NGS data set, demonstrating that index hopping had occurred and could therefore not be neglected. Index hopping could lead to barcode switching between samples so that reads would not be demultiplexed accurately, which inevitably leads to a wrong assignment of sequences to samples. The present results clearly indicate that an index hopping threshold should be mandatory for all NGS studies, including targeted amplicon sequencing. The use of the read counts of the index combinations of control samples to define the threshold has proven successful in this study. It is therefore a promising approach that can also be used in other studies. One alternative for assessing the effect of index hopping are spike-in controls of a validated reference DNA sample of alien species to estimate the effect of index hopping and data processing^54^. In particular, the use of different spike-in controls with known reference DNA fragments in one run is also suitable for dual indexing approaches. Working with unique dual indexes can be considered, provided the number of samples is small enough and the costs incurred play a minor role. If this is not possible, it is imperative to use controls (spike-in controls or for threshold definition) to determine the extent of index hopping and to take this into account when analysing the data set. Although the data set of this study refers to TAS, it was shown that index hopping as such is a critical problem, so that it can also be linked to other sequencing approaches, such as whole genome or transcriptome sequencing. In diet metabarcoding one has often to deal with small amounts of DNA that diminish even further during digestion. The same holds true for biodiversity studies when it comes to the detection of rare species. For this reason, it is particularly important to differentiate between small read numbers that are an effect of index hopping and those that are real products of low quantity food or species DNA. To date, index hopping rates are typically not estimated or not provided in publications. This makes it difficult to evaluate the outcomes of studies in the literature. In the rapidly evolving field of NGS analysis many different software tools and pipelines are available. The main objective of the present approach for the bioinformatic analysis of NGS data was to achieve validated results that allow for an individual based analysis. Here, we provide a comprehensive description, a protocol, of sample processing. In doing this, the nested metabarcoding approach was found to be a very suitable approach for analysing large numbers of individual samples so long as a tailored demultiplexing method was used. In principle, the protocol used here can also be implemented for dual indexing approaches, with the exception that the demultiplexing step (see Fig. 2, step 3) is omitted. For nested metabarcoding approaches, it is also possible to adapt the input files for demultiplexing to your own needs. If other index combinations are used than in this study, then only the index sequences in the csv file would have to be changed in the respective plate position (see supplements for more details). We recommend a collaboration with experts within this specialized field regarding the wide range of software applications for bioinformatic analysis, often requiring the use of programming languages and scripting. The present results clearly indicate that accurate data processing depends on both the parameters used and the setting of a threshold for index hopping. Furthermore, many different aspects can already influence the data set in advance and thus be important for data processing, such as library preparation, general sample handling, flow cell loading and thus the sequencing run quality and also which sequencing platform was used. Information on these aspects can therefore be of decisive importance in addition to the software tools and their settings. Moreover, our work would argue that a detailed description of raw data handling as well as the bioinformatic analysis tools and settings are essential in all future studies, as this will strengthen our ability to understand and compare NGS-derived (ecological) data sets.

## Supplementary Information

Supplementary Information 1.

Supplementary Information 2.

## Data Availability

Further information can be found in the supplements of the online version of this article. The specifically written bash script of this study and some more information on how to run it is also available in the supplements.
