# Supplementary material for: Handling of targeted amplicon sequencing data focusing on index hopping and demultiplexing using a nested metabarcoding approach in ecology
Source: Sci Rep. 2021 Sep 30;11:19510. doi: 10.1038/s41598-021-98018-4 (PMC8484467; doi:10.1038/s41598-021-98018-4)
Supplement: Supplementary file 1 — Supplementary Information 1. [file 41598_2021_98018_MOESM1_ESM.pdf]

Supplementary Information

'Handling of targeted amplicon sequencing data focusing on index hopping and demultiplexing using a nested metabarcoding approach in ecology'

Yasemin Guenay-Greunke[1,2], David A. Bohan[3], Michael Traugott[1] and Corinna Wallinger[1,2]

[1]Applied Animal Ecology, Department of Zoology, University of Innsbruck, Technikerstraße 25, 6020 Innsbruck, Austria.

[2] Institute of Interdisciplinary Mountain Research, IGF, Austrian Academy of Sciences, Technikerstraße 21a, 6020 Innsbruck, Austria.

[3]Agroécologie, AgroSup Dijon, INRAE, Université Bourgogne Franche-Comté, F-21000 Dijon, France.

ORCID

Yasemin Günay-Greunke	*https://orcid.org/0000-0002-7054-3380*
David A. Bohan	*https://orcid.org/0000-0001-5656-775X*
Michael Traugott	*https://orcid.org/0000-0001-9719-5059*
Corinna Wallinger	*https://orcid.org/0000-0002-7153-9690*

Journal: Scientific Reports

Type of article: Research article

Number of words in the abstract: 223

Word count for main text: ~ 5,000 (not including Abstract, Methods, References and figure legends)

Corresponding author:

Yasemin Guenay-Greunke: yasemin.guenay-greunke.science@outlook.com

SUPPLEMENTARY TABLES

Table S1: List of used modified *ITS2* primers in the first PCR: Nextera adapter binding site underlined, sequencing primer binding site is depicted in lowercase letters, indexes are in lowercase letters and highlighted in bold and the amplicon primer sequence in uppercase.

Primer name	Adapter binding site, sequencing primer binding site, inner index region, plant primer sequence (5' → 3')	Direction	Nextera index ID and sequence
UniPlantF2_S513	tcgtcggcagcgtcagatgtgtataagagacag**tcgactag**GGCACGYCTGYBTGG	forward	S513: tcgactag
UniPlantF2_S515	tcgtcggcagcgtcagatgtgtataagagacag**ttctagct**GGCACGYCTGYBTGG	forward	S515: ttctagct
UniPlantF2_S516	tcgtcggcagcgtcagatgtgtataagagacag**cctagagt**GGCACGYCTGYBTGG	forward	S516: cctagagt
UniPlantF2_S517	tcgtcggcagcgtcagatgtgtataagagacag**gcgtaaga**GGCACGYCTGYBTGG	forward	S517: gcgtaaga
UniPlantF2_S518	tcgtcggcagcgtcagatgtgtataagagacag**ctattaag**GGCACGYCTGYBTGG	forward	S518: ctattaag
UniPlantF2_S520	tcgtcggcagcgtcagatgtgtataagagacag**aaggctat**GGCACGYCTGYBTGG	forward	S520: aaggctat
UniPlantF2_S521	tcgtcggcagcgtcagatgtgtataagagacag**gagcctta**GGCACGYCTGYBTGG	forward	S521: gagcctta
UniPlantF2_S522	tcgtcggcagcgtcagatgtgtataagagacag**ttatgcga**GGCACGYCTGYBTGG	forward	S522: ttatgcga
UniplantR_N716	gtctcgtgggctcggagatgtgtataagagacag**tagcgagt**CCCGHYTGAYYTGRGGTCDC	reverse	N716: tagcgagt
UniplantR_N718	gtctcgtgggctcggagatgtgtataagagacag**gtagctcc**CCCGHYTGAYYTGRGGTCDC	reverse	N718: gtagctcc
UniplantR_N719	gtctcgtgggctcggagatgtgtataagagacag**tactacgc**CCCGHYTGAYYTGRGGTCDC	reverse	N719: tactacgc
UniplantR_N720	gtctcgtgggctcggagatgtgtataagagacag**aggctccg**CCCGHYTGAYYTGRGGTCDC	reverse	N720: aggctccg
UniplantR_N721	gtctcgtgggctcggagatgtgtataagagacag**gcagcgta**CCCGHYTGAYYTGRGGTCDC	reverse	N721: gcagcgta
UniplantR_N722	gtctcgtgggctcggagatgtgtataagagacag**ctgcgcat**CCCGHYTGAYYTGRGGTCDC	reverse	N722: ctgcgcat
UniplantR_N723	gtctcgtgggctcggagatgtgtataagagacag**gagcgcta**CCCGHYTGAYYTGRGGTCDC	reverse	N723: gagcgcta
UniplantR_N724	gtctcgtgggctcggagatgtgtataagagacag**cgctcagt**CCCGHYTGAYYTGRGGTCDC	reverse	N724: cgctcagt
UniplantR_N726	gtctcgtgggctcggagatgtgtataagagacag**gtcttagg**CCCGHYTGAYYTGRGGTCDC	reverse	N726: gtcttagg
UniplantR_N727	gtctcgtgggctcggagatgtgtataagagacag**actgatcg**CCCGHYTGAYYTGRGGTCDC	reverse	N727: actgatcg
UniplantR_N728	gtctcgtgggctcggagatgtgtataagagacag**tagctgca**CCCGHYTGAYYTGRGGTCDC	reverse	N728: tagctgca
UniplantR_N729	gtctcgtgggctcggagatgtgtataagagacag**gacgtcga**CCCGHYTGAYYTGRGGTCDC	reverse	N729: gacgtcga

Table S2: List of used Illumina index adapter sequences in the second PCR: flow cell binding site is depicted in lowercase letters; indexes are in lowercase letters and highlighted in bold and the adapter primer sequence in uppercase.

**General adapter information:**		
Index 1 (i7) Adapters: caagcagaagacggcatacgagat [i7] GTCTCGTGGGCTCGG		
Index 2 (i5) Adapters: aatgatacggcgaccaccgagatctacac [i5] TCGTCGGCAGCGTC		
**Adapter name**	**Flow cell binding site, outer index region, adapter primer sequence (5' → 3')**	**Nextera index ID and sequence**
Nxt_N702	caagcagaagacggcatacgagat**ctagtacg**GTCTCGTGGGCTCGG	N702: ctagtacg
Nxt_N703	caagcagaagacggcatacgagat**ttctgcct**GTCTCGTGGGCTCGG	N703: ttctgcct
Nxt_N704	caagcagaagacggcatacgagat**gctcagga**GTCTCGTGGGCTCGG	N704: gctcagga
Nxt_N705	caagcagaagacggcatacgagat**aggagtcc**GTCTCGTGGGCTCGG	N705: aggagtcc
Nxt_S502	aatgatacggcgaccaccgagatctacac**ctctctat**TCGTCGGCAGCGTC	S502: ctctctat
Nxt_S503	aatgatacggcgaccaccgagatctacac**tatcctct**TCGTCGGCAGCGTC	S503: tatcctct
Nxt_S505	aatgatacggcgaccaccgagatctacac**gtaaggag**TCGTCGGCAGCGTC	S505: gtaaggag
Nxt_S506	aatgatacggcgaccaccgagatctacac**actgcata**TCGTCGGCAGCGTC	S506: actgcata
Nxt_S507	aatgatacggcgaccaccgagatctacac**aaggagta**TCGTCGGCAGCGTC	S507: aaggagta
Nxt_S508	aatgatacggcgaccaccgagatctacac**ctaagcct**TCGTCGGCAGCGTC	S508: ctaagcct

Table S3: Overview of the read counts received per plate sample as raw data, after the merging step (step 2) and after the demultiplexing step (step 3), as well as their data loss between the respective steps from raw data to step 2 and from step 2 to 3.

Sequencing lane and plate		Read counts of the raw data	Read counts after step 2	% of data loss per plate sample after step 2	Read counts after step 2 and 3	% of data loss per plate sample between step 2 and 3
Lane 1	Plate 1	6,713,041	6,645,645	1.00	6,325,643	4.82
	Plate 2	5,932,189	5,868,158	1.08	5,609,301	4.41
	Plate 3	5,872,476	5,801,292	1.21	5,538,205	4.53
	Plate 4	5,935,608	5,867,235	1.15	5,632,861	3.99
	Plate 5	6,084,160	6,042,819	0.68	5,789,051	4.20
	Plate 6	6,756,697	6,696,464	0.89	6,400,943	4.41
	Plate 7	6,761,259	6,704,141	0.84	6,324,072	5.67
	Plate 8	6,378,446	6,296,597	1.28	6,022,317	4.36
	Plate 9	5,945,599	5,886,989	0.99	5,567,976	5.42
	Plate 10	6,723,619	6,637,401	1.28	6,297,974	5.11
	Plate 11	7,855,719	7,775,498	1.02	7,297,045	6.15
	Plate 12	6,729,046	6,659,800	1.03	6,370,226	4.35
Lane 2	Plate 13	6,126,873	6,064,493	1.02	4,842,160	20.16
	Plate 14	7,169,516	7,088,091	1.14	5,445,140	23.18
	Plate 15	5,980,536	5,914,990	1.10	4,731,677	20.01
	Plate 16	6,419,771	6,345,952	1.15	5,167,345	18.57
	Plate 17	5,505,974	5,415,150	1.65	4,287,737	20.82
	Plate 18	9,187,035	9,052,475	1.46	7,076,954	21.82
	Plate 19	6,338,858	6,248,641	1.42	5,436,216	13.00
	Plate 20	6,967,920	6,860,084	1.55	5,528,801	19.41
	Plate 21	6,464,257	6,398,173	1.02	5,296,050	17.23
	Plate 22	7,460,730	7,362,073	1.32	5,969,024	18.92
	Plate 23	7,519,296	7,404,937	1.52	5,894,229	20.40
	Plate 24	7,239,640	7,109,222	1.80	5,846,375	17.76

SUPPLEMENTARY FIGURES

Sample		i7 indices											
		N716	N718	N719	N720	N721	N722	N723	N724	N726	N727	N728	N729
i5 indices	S513	A1	A2	A3	A4	A5	A6	A7	A8	A9	A10	A11	A12
	S515	B1	B2	B3	B4	B5	B6	B7	B8	B9	B10	B11	B12
	S516	C1	C2	C3	C4	C5	C6	C7	C8	C9	C10	C11	C12
	S517	D1	D2	D3	D4	D5	D6	D7	D8	D9	D10	D11	D12
	S518	E1	E2	E3	E4	E5	E6	E7	E8	E9	E10	E11	E12
	S520	F1	F2	F3	F4	F5	F6	F7	F8	F9	F10	F11	F12
	S521	G1	G2	G3	G4	G5	G6	G7	G8	G9	G10	G11	G12
	S522	H1	H2	H3	H4	H5	H6	H7	H8	H9	H10	H11	H12

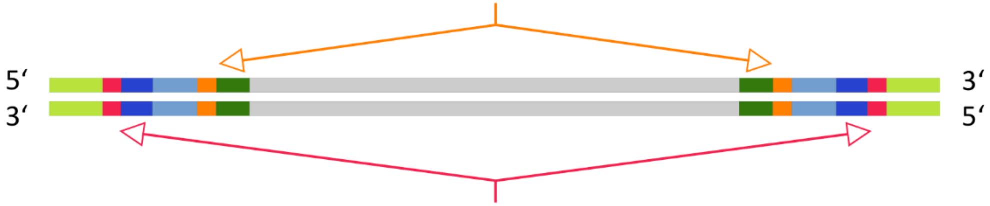

Plate		i7 indices			
		N702	N703	N704	N705
i5 indices	S502	Plate 1	Plate 7	Plate 13	Plate 19
	S503	Plate 2	Plate 8	Plate 14	Plate 20
	S505	Plate 3	Plate 9	Plate 15	Plate 21
	S506	Plate 4	Plate 10	Plate 16	Plate 22
	S507	Plate 5	Plate 11	Plate 17	Plate 23
	S508	Plate 6	Plate 12	Plate 18	Plate 24

Figure S1: The tagging scheme of the nested metabarcoding approach used for this study.

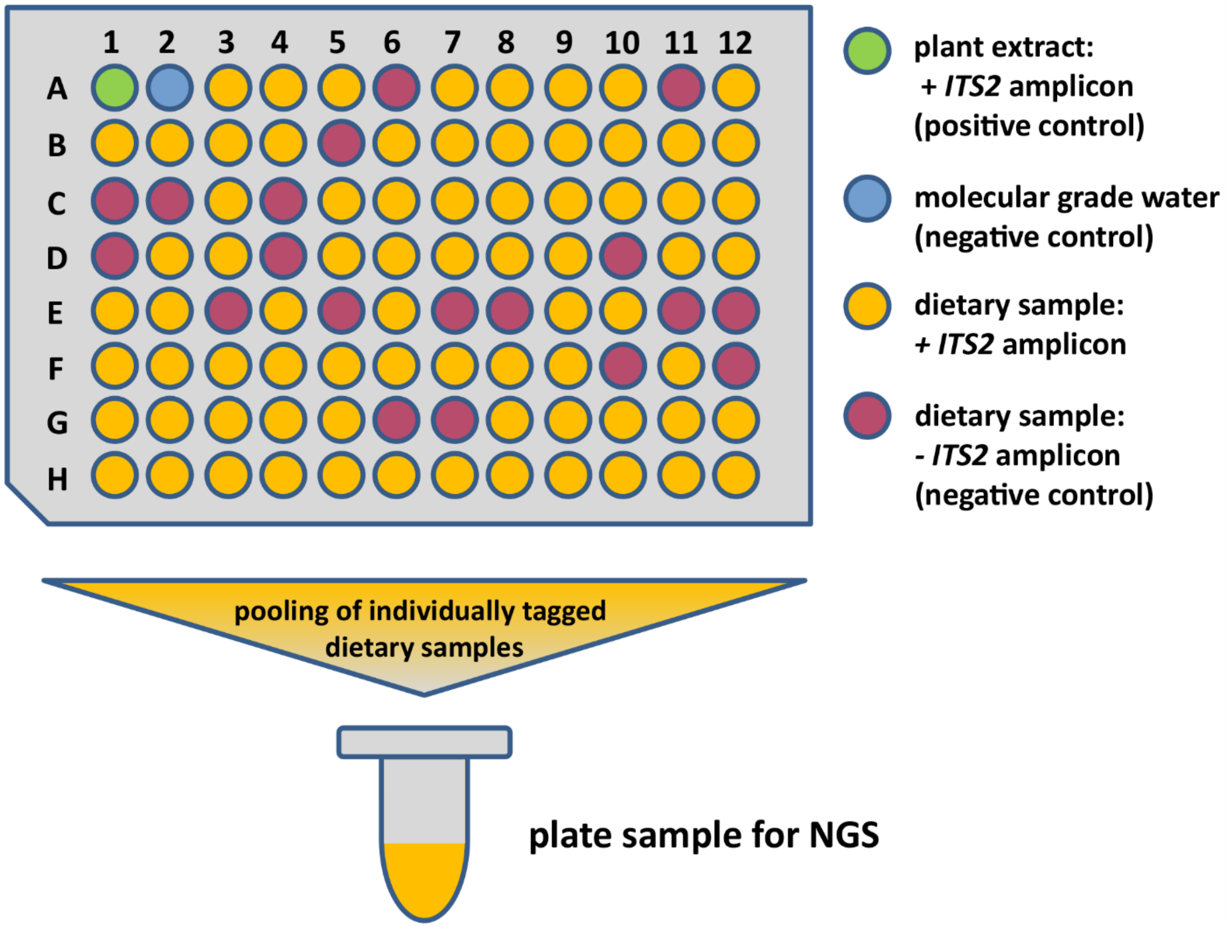

Figure S2: Library preparation for plate samples; positive and negative controls were not sent to NGS, therefore serving as quality control for index hopping; for each of the 24 plate controls and dietary samples were distributed differently across each plate.

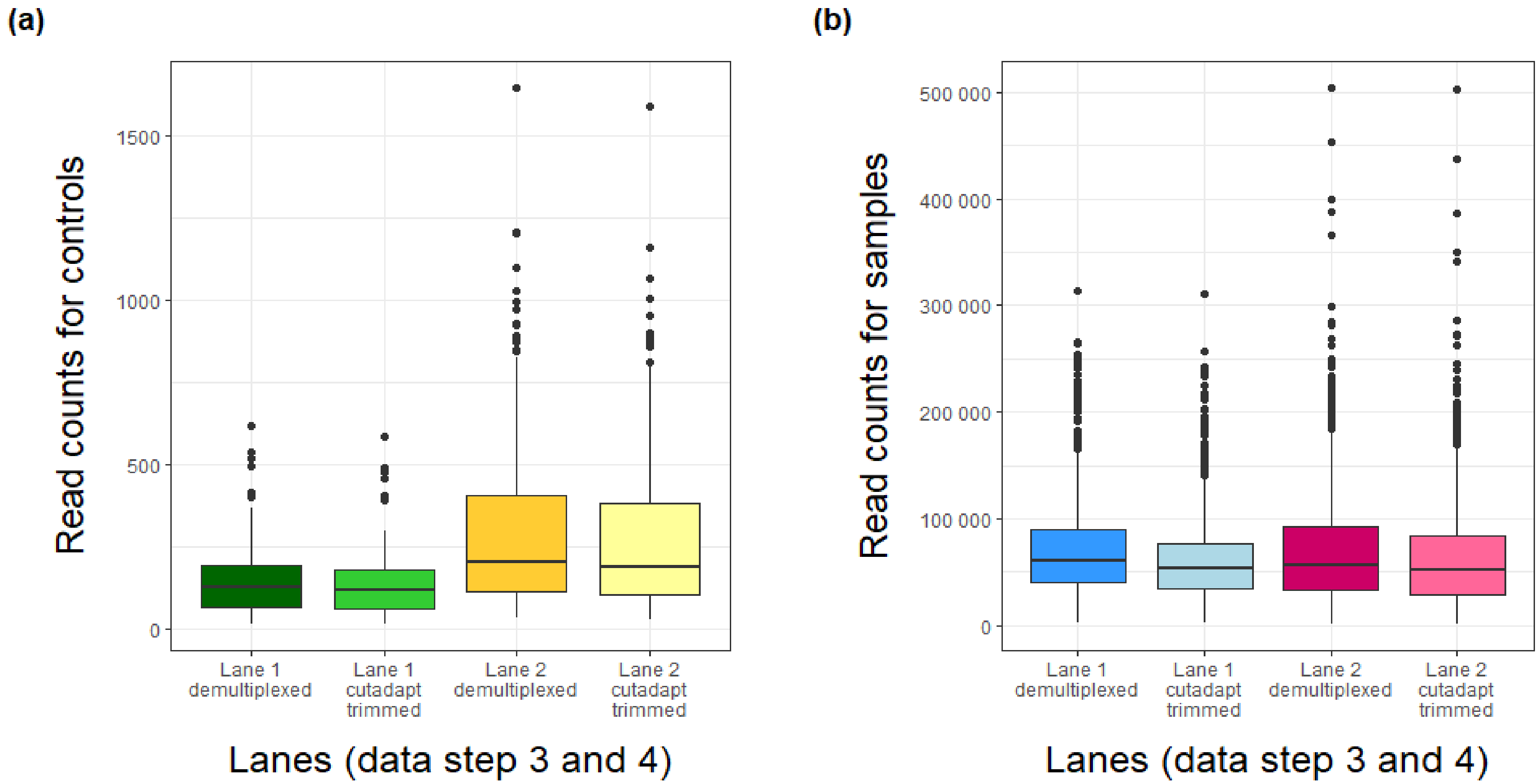

Figure S3: **Comparison of read counts for (a) the 370 control samples and (b) the 1920 dietary samples between lane 1 and 2 after the demultiplexing and adapter trimming step.**
