# Supplementary material for: Handling of targeted amplicon sequencing data focusing on index hopping and demultiplexing using a nested metabarcoding approach in ecology
Source: Sci Rep. 2021 Sep 30;11:19510. doi: 10.1038/s41598-021-98018-4 (PMC8484467; doi:10.1038/s41598-021-98018-4)
Supplement: Supplementary file 2 — Supplementary Information 2. [file 41598_2021_98018_MOESM2_ESM.zip › Demultiplexing_How_to_for_bash_script.docx]

How to run the bash script ‘plate2pos_v2_demultiplexing.sh’

**Requirements:**

At least bash version 4.x.

**Usage:**

General note on how to run the bash script for demultiplexing:

Three files are stored within a folder:

1. the bash script itself with the name 'plate2pos_v2_demultiplexing.sh';
2. a csv file (here with the name 'position_indexes.csv'), which contains the individual plate position of the 96-well plate with the subsequent index combinations of the inner indexes per line; if you use different index combinations, make changes to this file; in this case the index sequences are then adapted to the combination used for each plate position and
3. a fastq file of the sequencer (here a test file with the name 'plate.fastq'), which has already created a fastq file based on the outer indexes, which in turn is to be read by the bash script.

The reads of each index combination are saved separately in a fastq file, which is given the original name of the fastq file with the addition of the plate position.

All of these newly generated, now demultiplexed files are saved in the same folder of these three files.

Command to execute the script within the Linux terminal: ./plate2pos_v2_demultiplexing.sh position_indexes.csv plate.fastq

Please check that the bash script is executable, otherwise run 'chmod -v +x plate2pos_v2_demultiplexing.sh'.

**Test sample set:**

We provide a test zip file containing the three files:

- plate2pos_v2_demultiplexing.sh (bash script)
- position_indexes.csv (csv file with inner index combinations)
- plate.fastq (fastq test data)

**Data processing with these test files:**

Depending on your computer system (CPUs), the data processing might take about one hour.
